# Supplementary figures and images for: Ninjurin 2, a Cell Adhesion Molecule and a Target of p53, Modulates Wild-Type p53 in Growth Suppression and Mutant p53 in Growth Promotion
Source: Cancers (Basel). 2024 Jan 4;16(1):229. doi: 10.3390/cancers16010229 (PMC10778559; doi:10.3390/cancers16010229)

Fig.1A

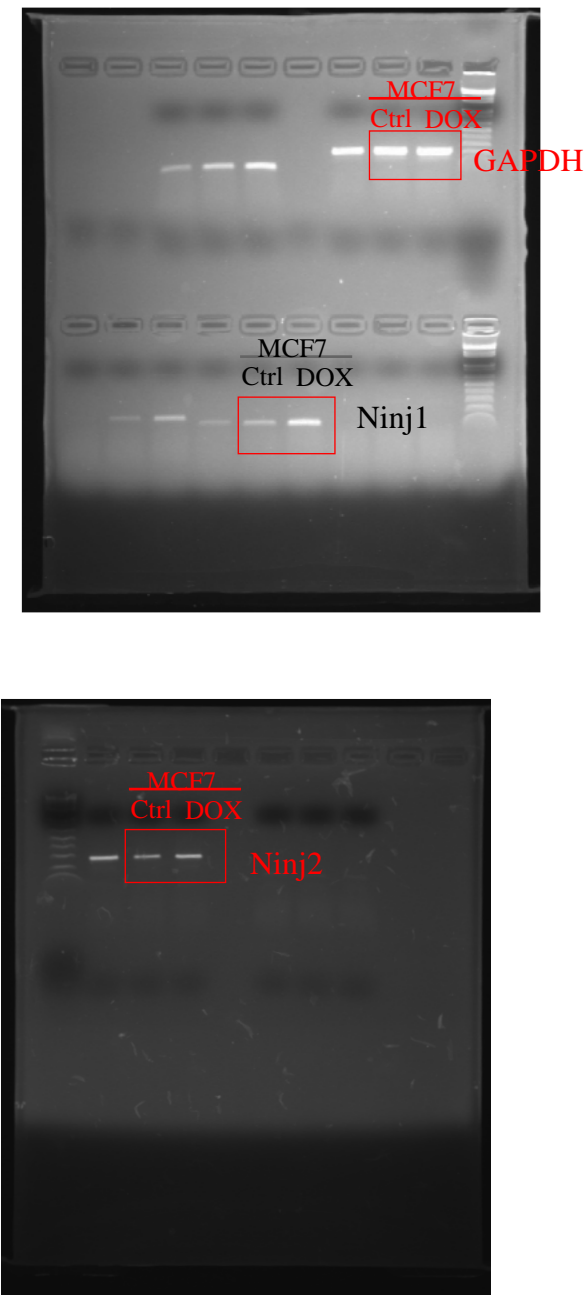

Fig1B

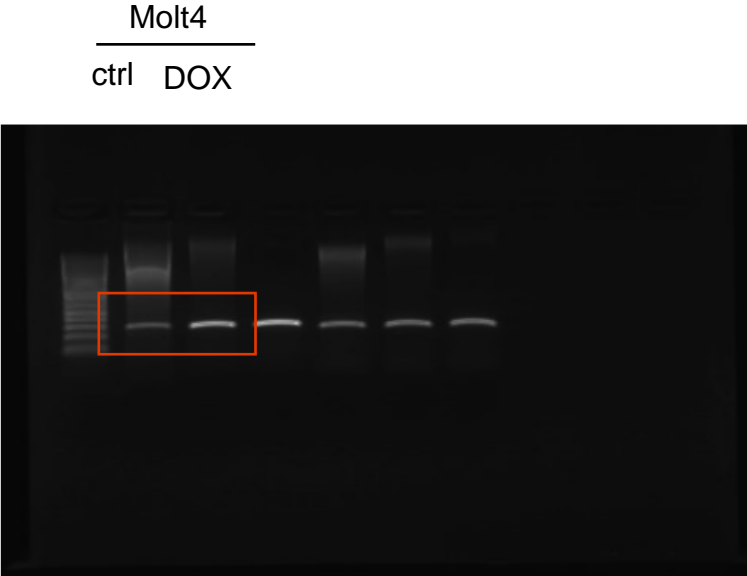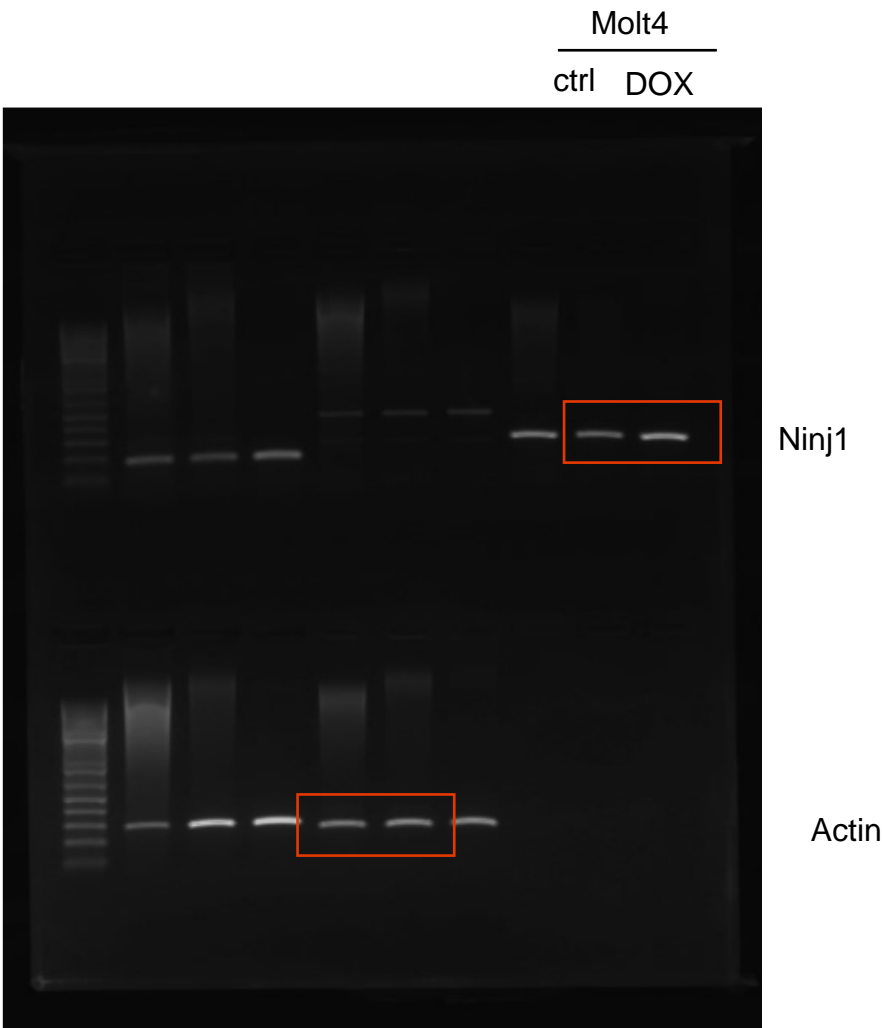

Fig. 1C

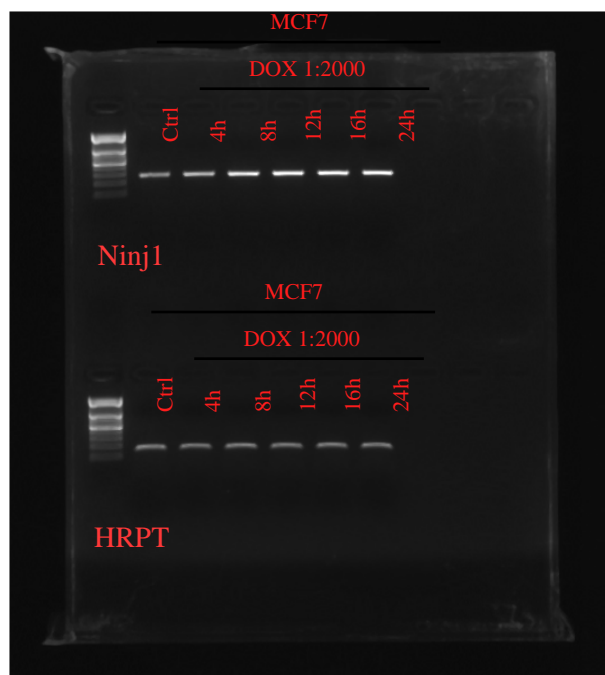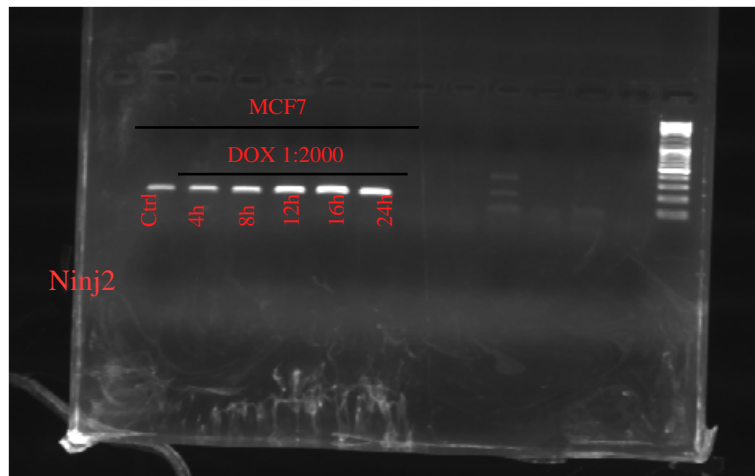

Fig. 1D

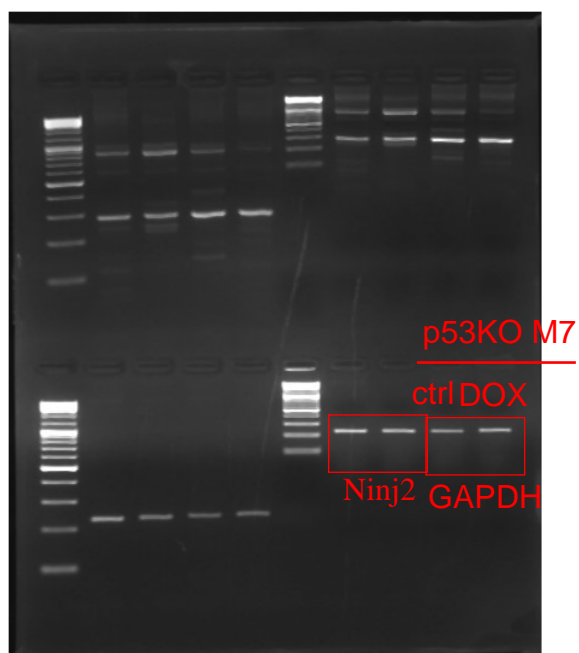

p53

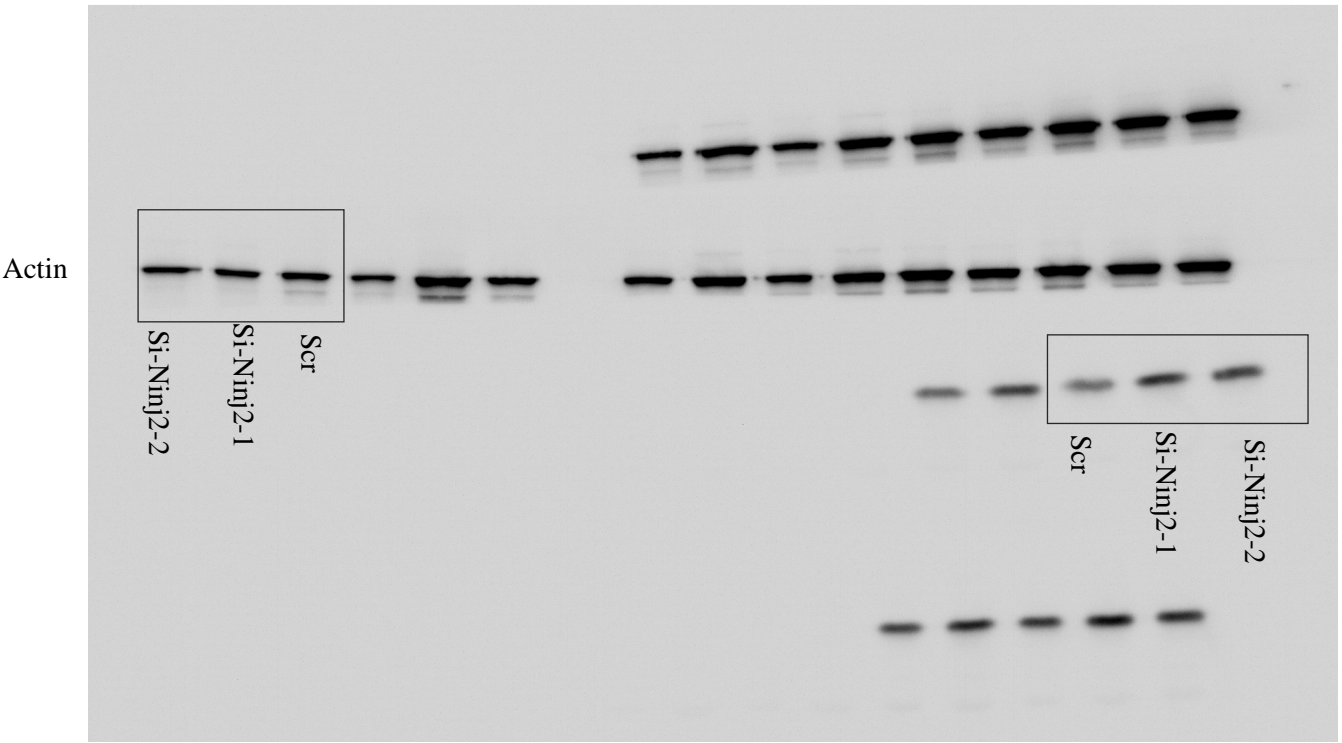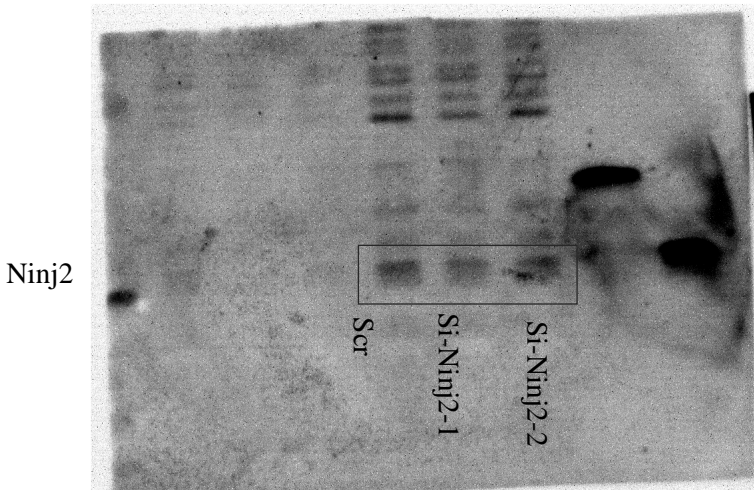

Fig. 1E

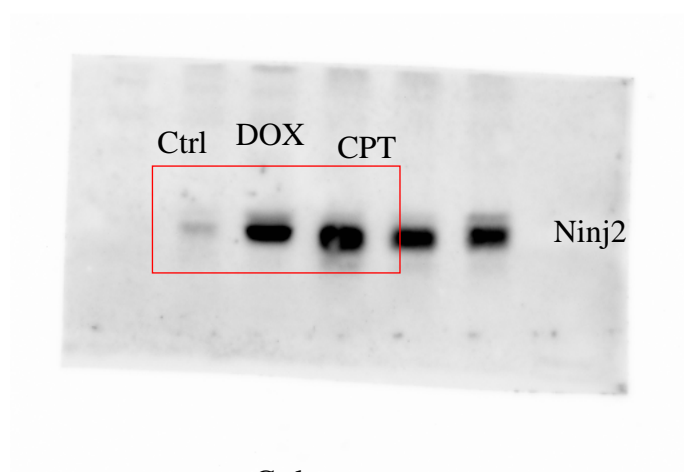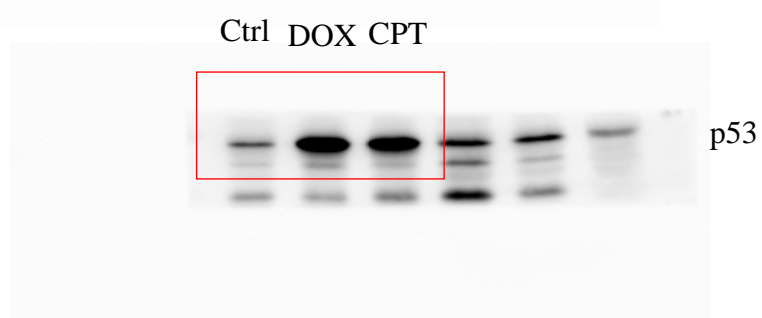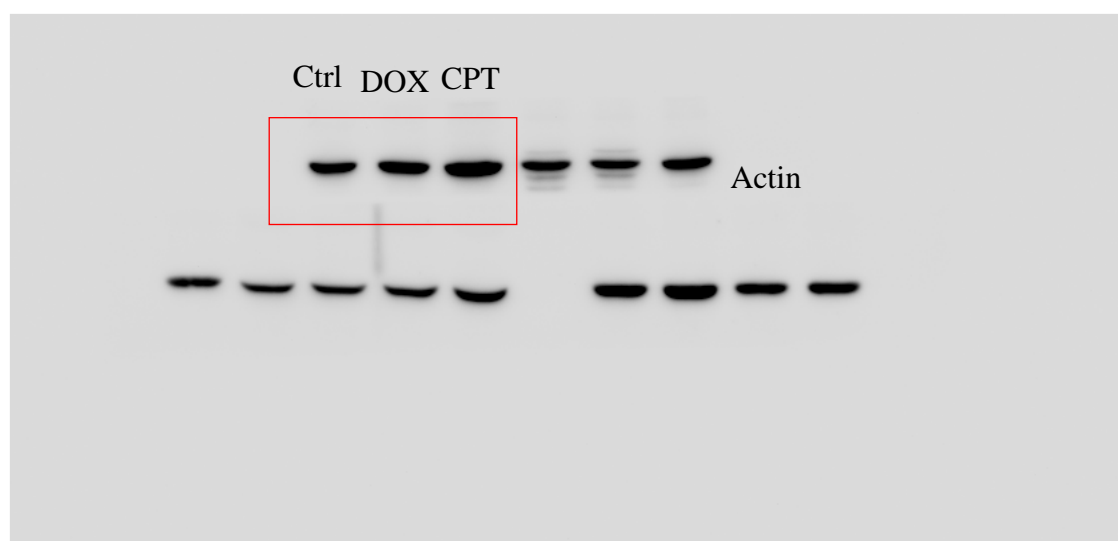

Fig. 1G

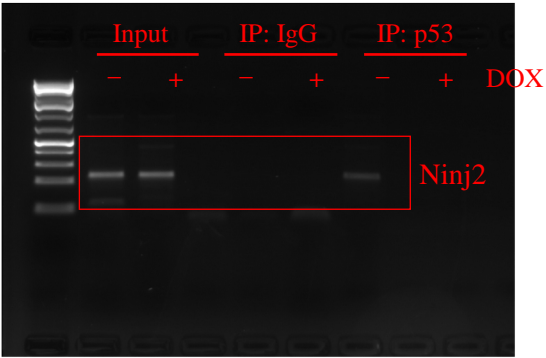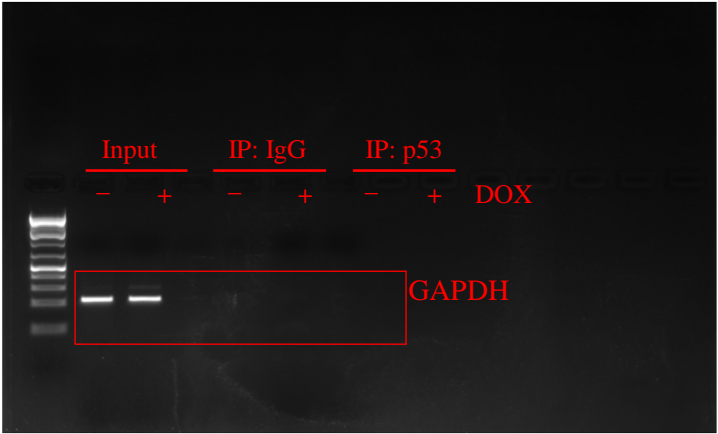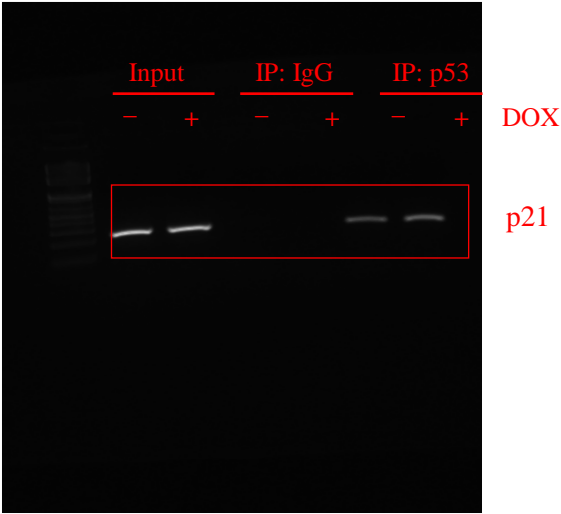

Fig. 2A

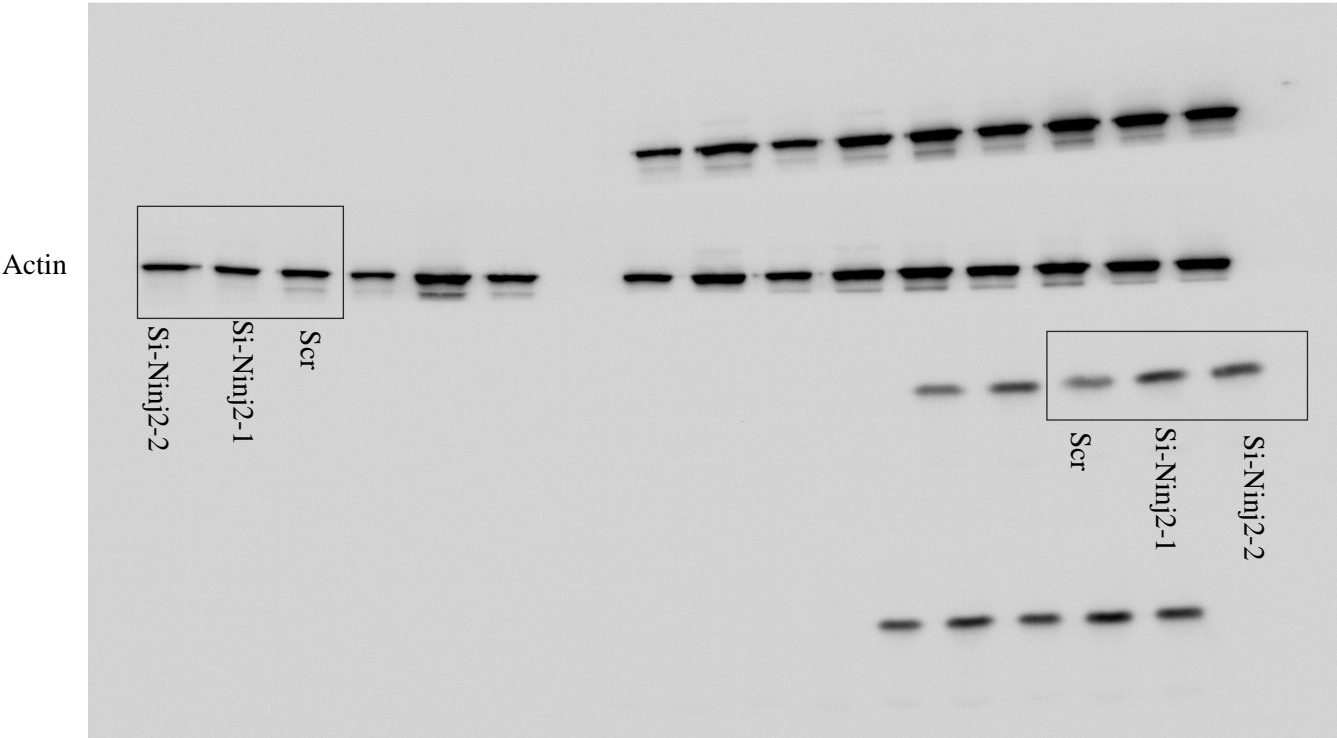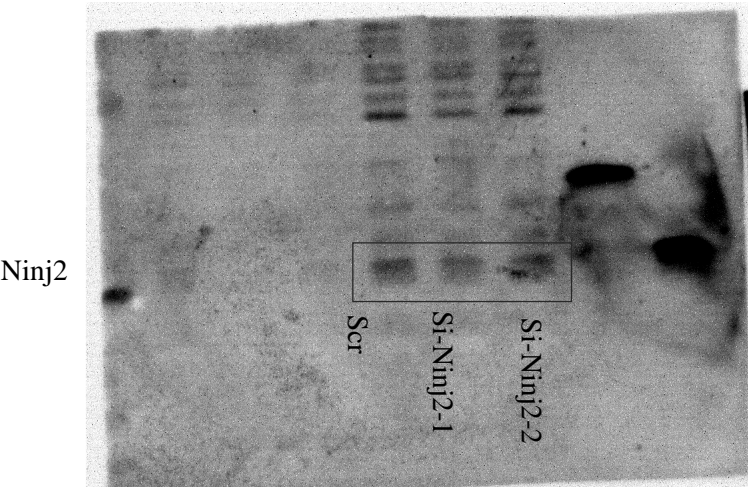

Fig. 2B

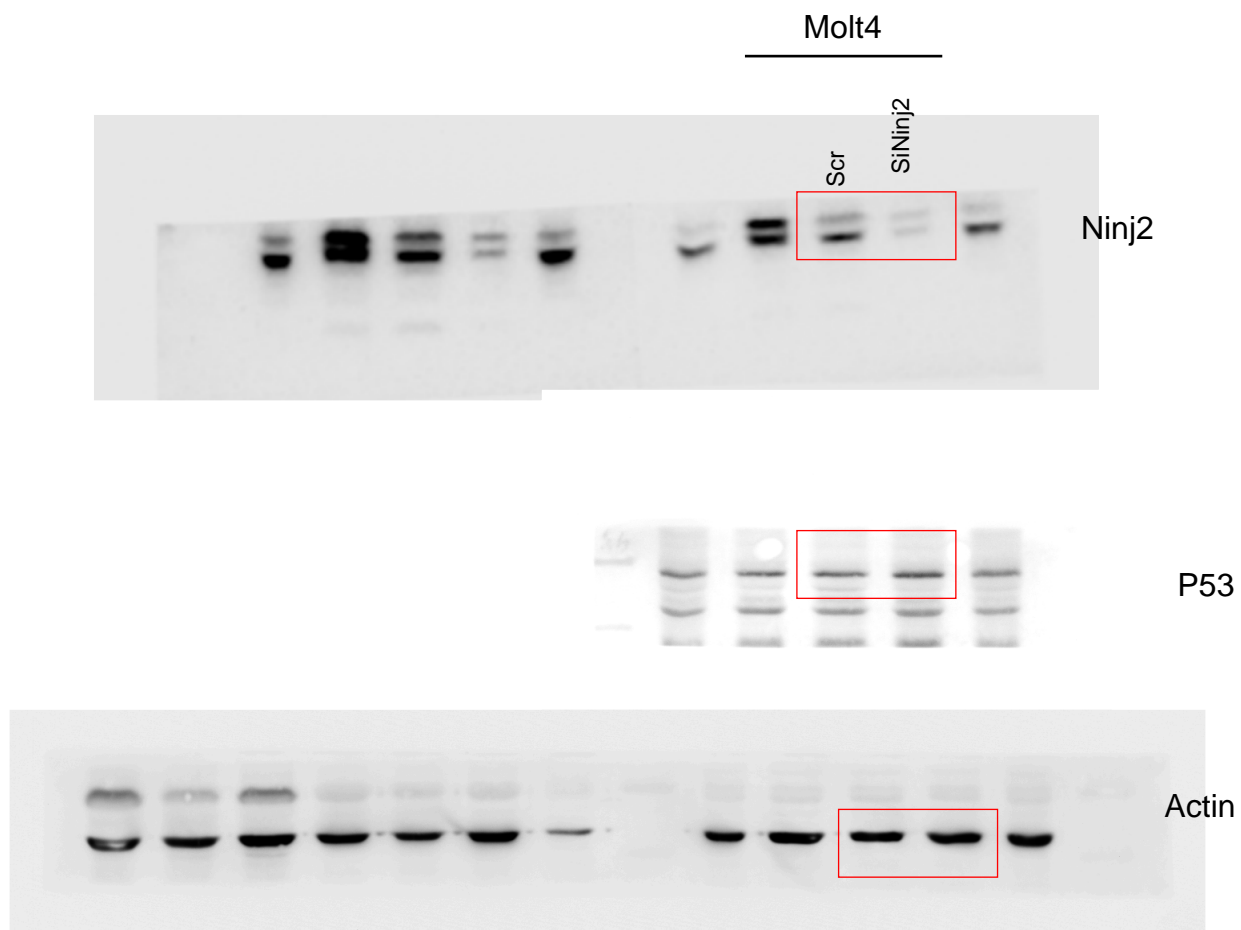

Fig2C

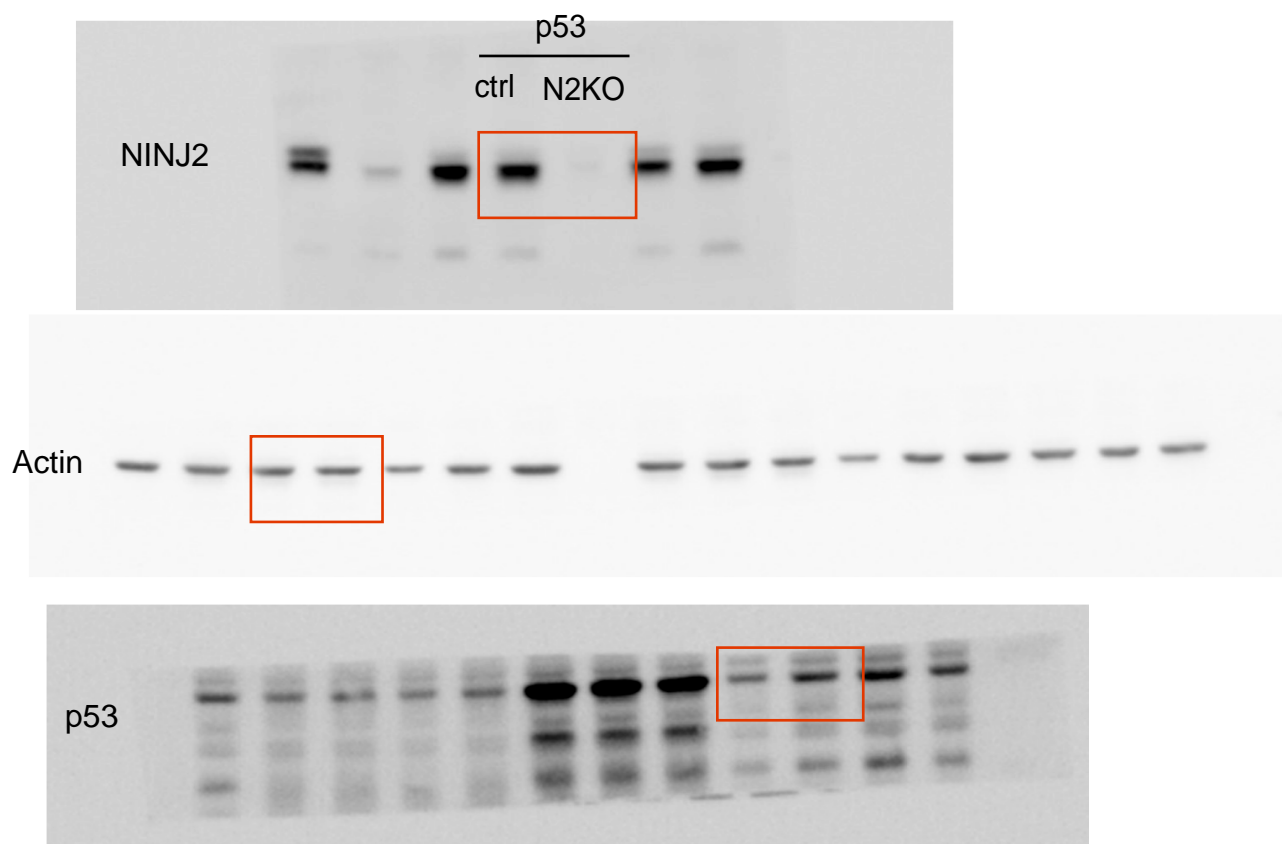

Fig. 2D

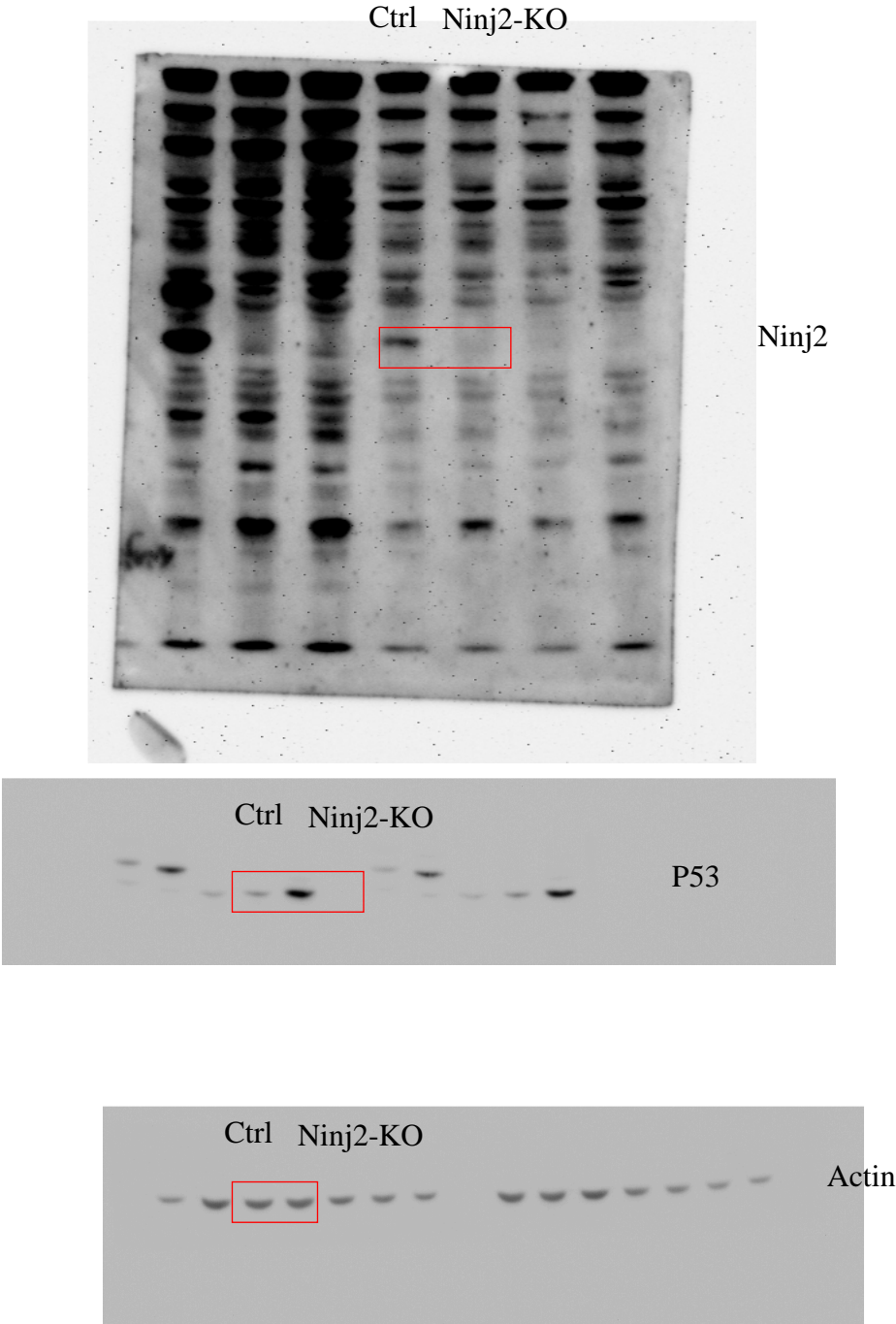

Fig2E

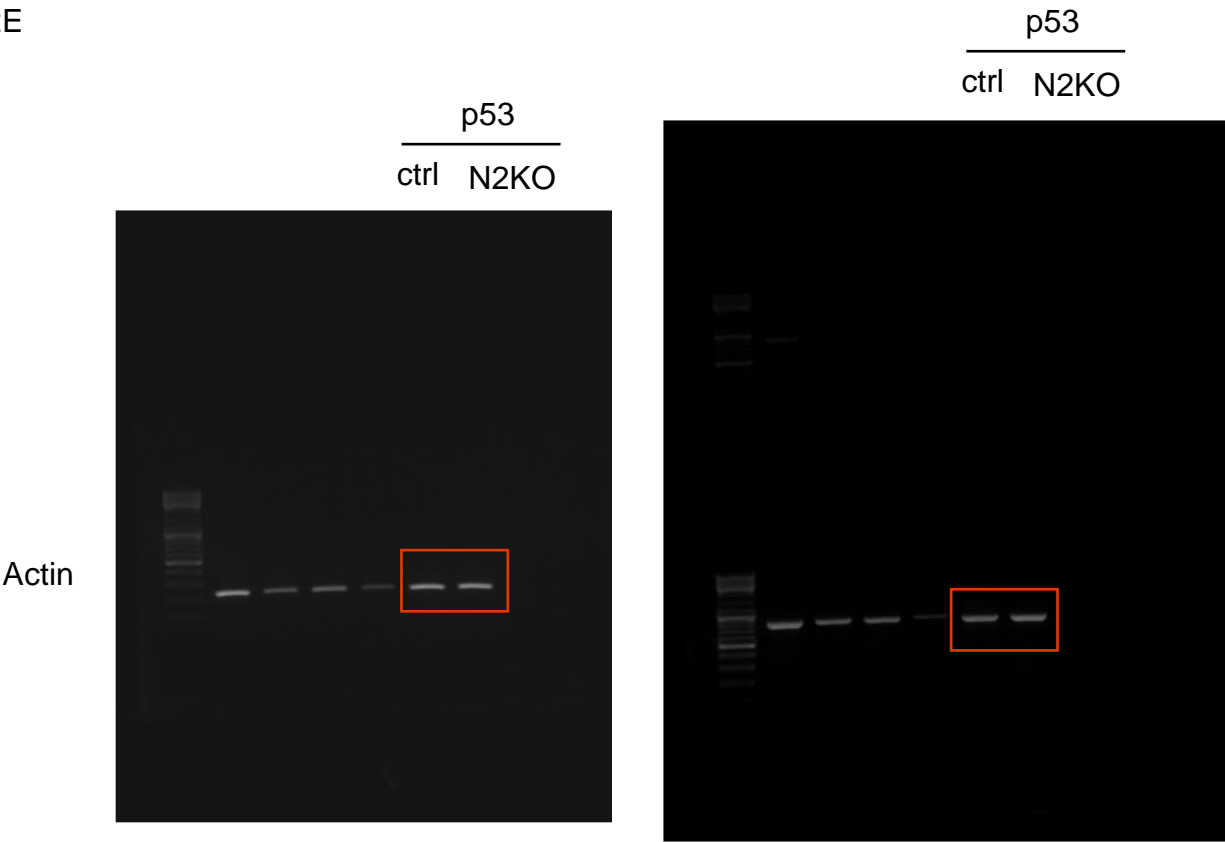

FIG. 2F

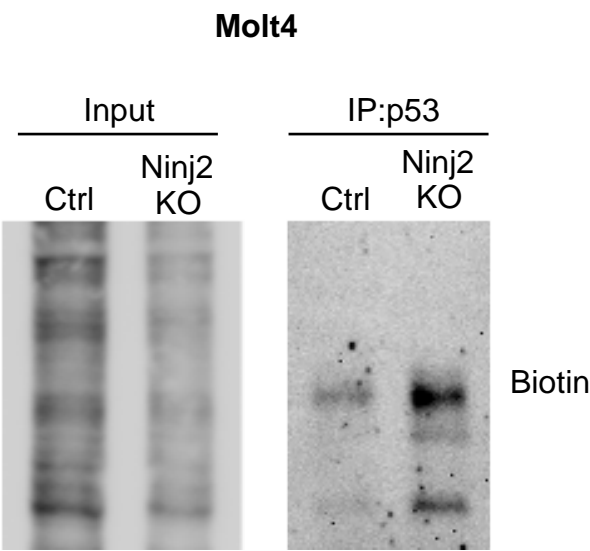

Fig. 3B

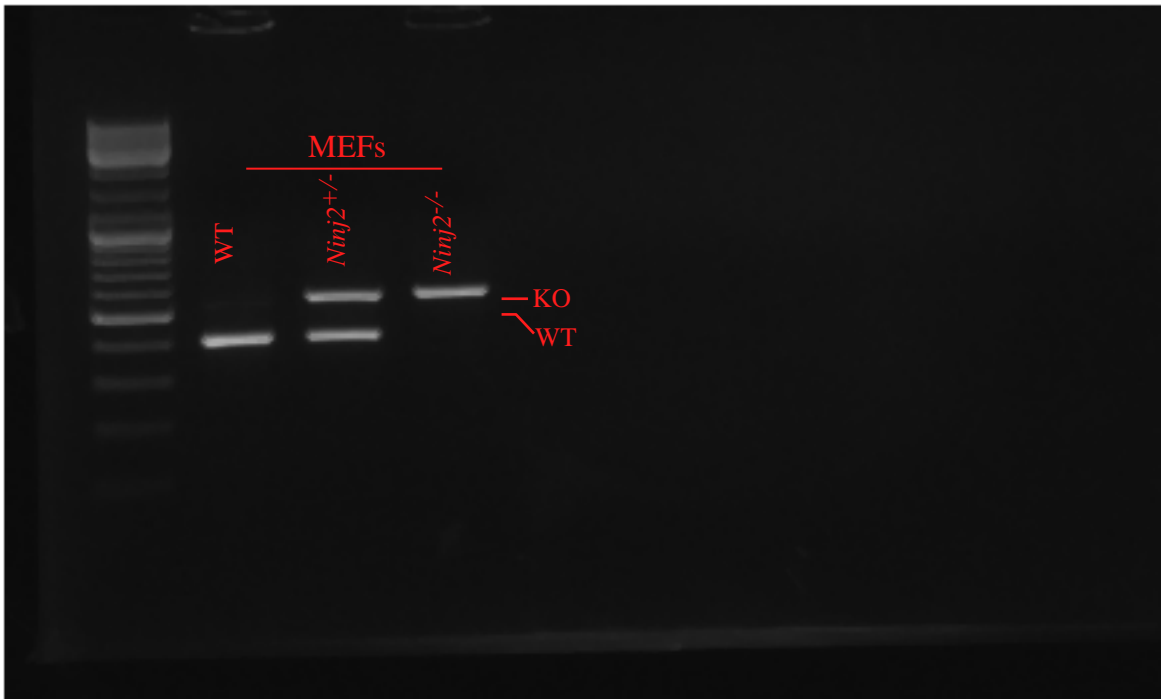

Fig. 3C

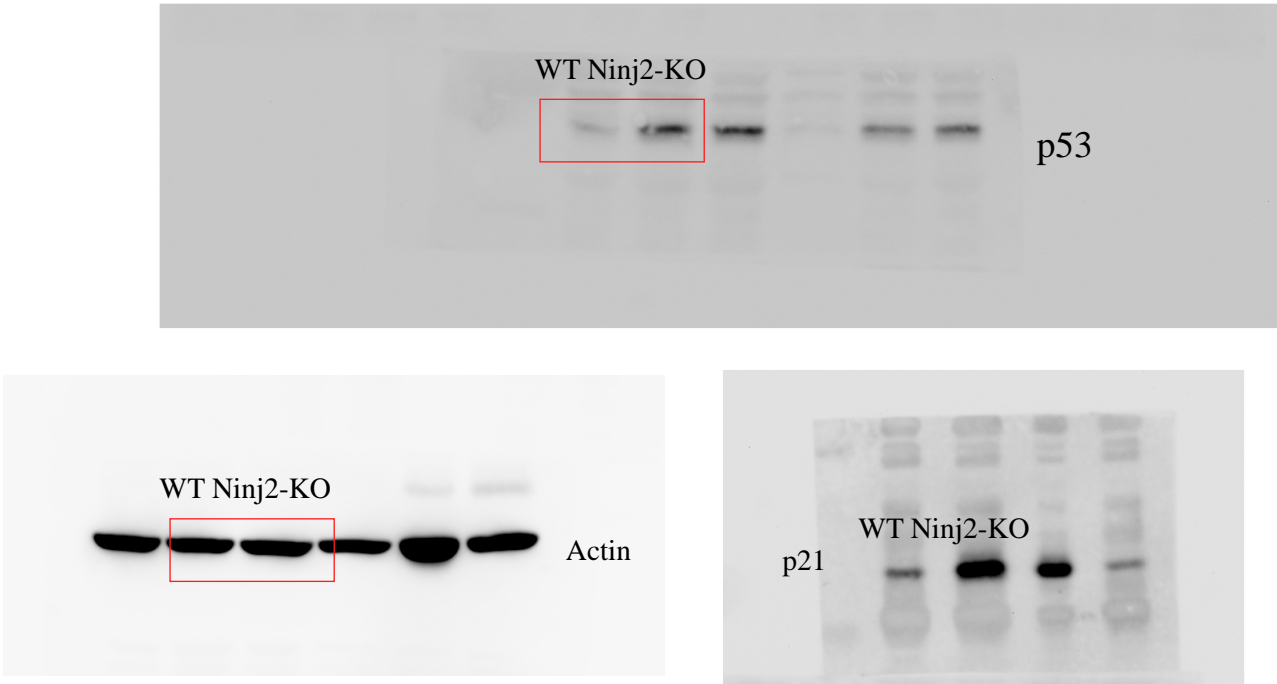

Figure 4D

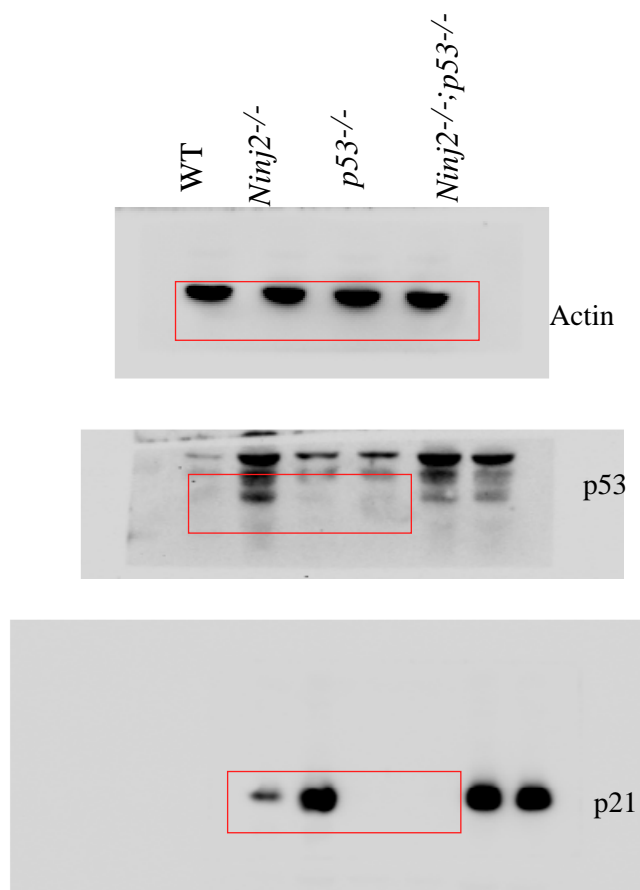

Fig. 5A

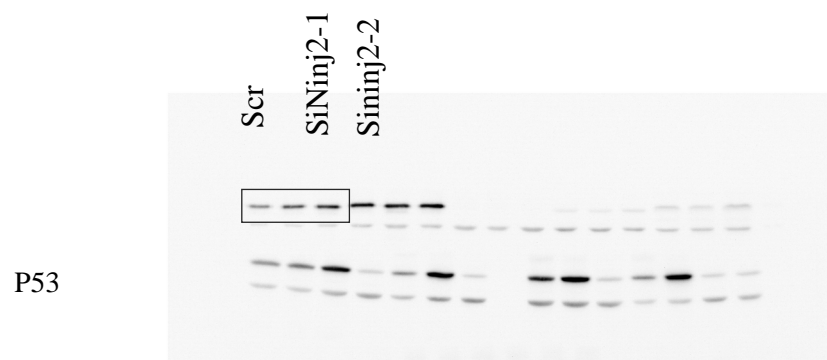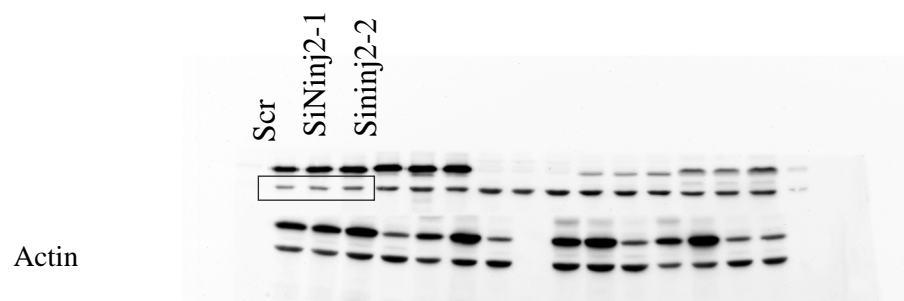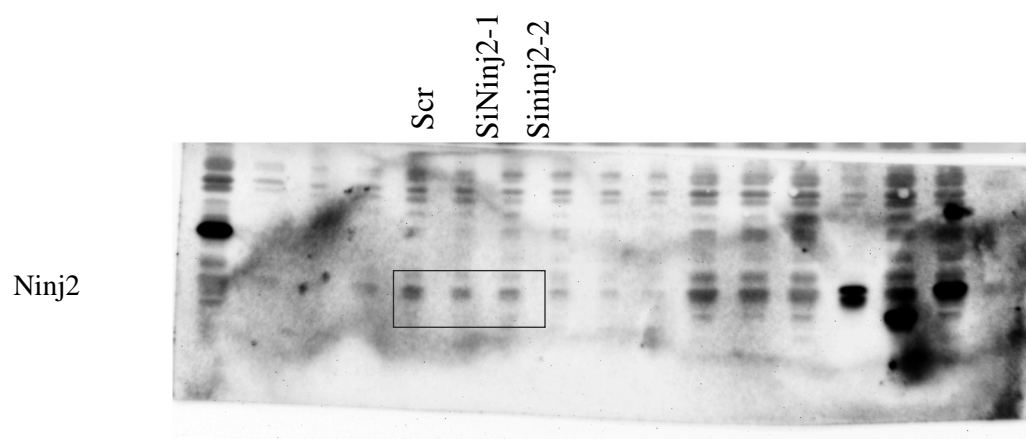

Fig 5B

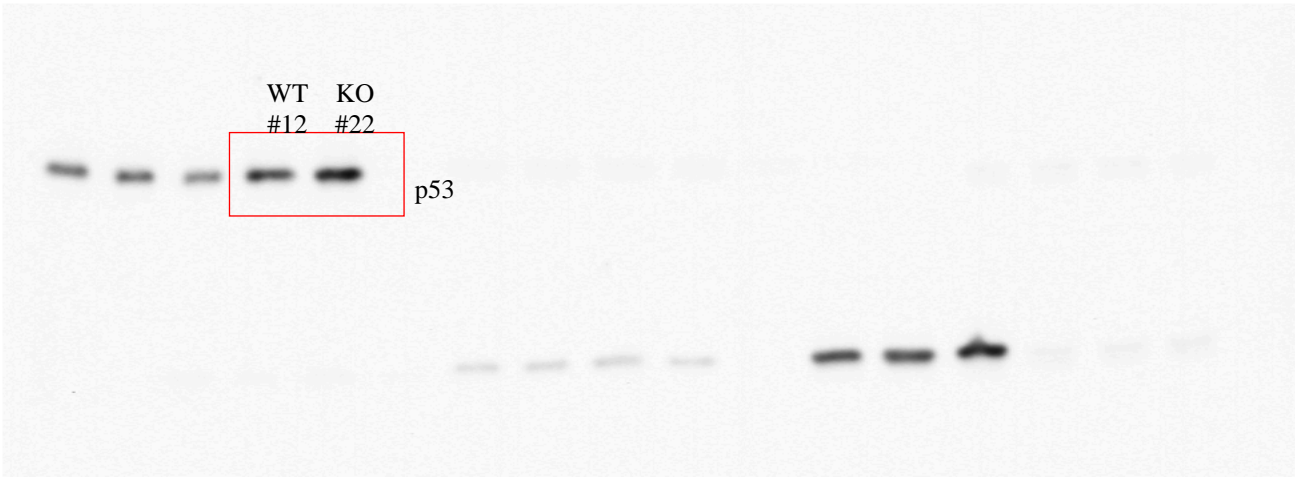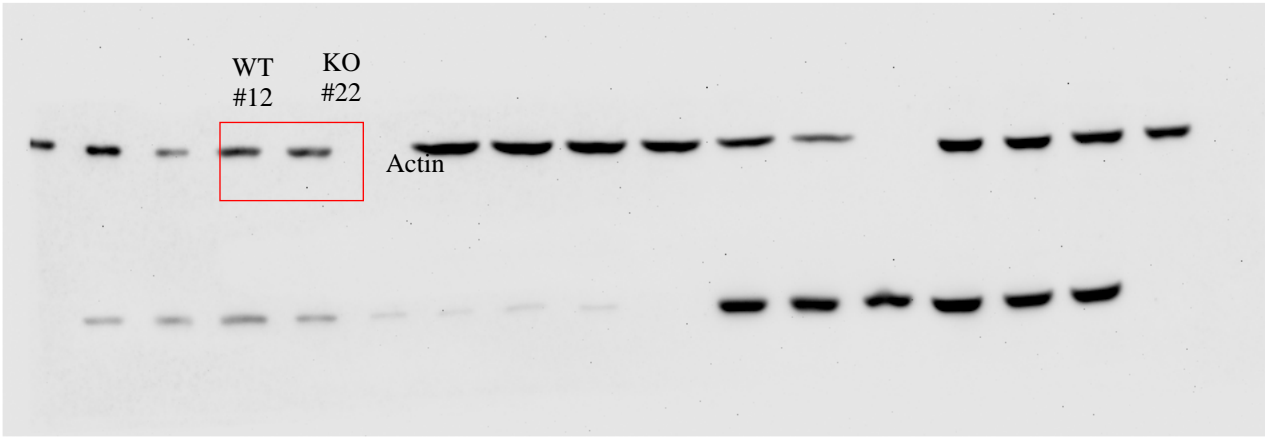

Supplement: Supplementary file 1 [file cancers-16-00229-s001.zip › cancers-2778595-supplementary.pdf]
